# Feminist Social Vision: Seeing Through the Lens of Marginalized Perceivers

**DOI:** 10.1177/10888683221126582

**Published:** 2022-10-11

**Authors:** Flora Oswald, Reginald B. Adams

**Affiliations:** 1 The Pennsylvania State University, University Park, USA

**Keywords:** social psychology, standpoint theory, social identity, vision science, face perception, body perception, intergroup relations

## Abstract

**Abstract:**

Social vision research, which examines, in part, how humans visually perceive social stimuli, is well-positioned to improve understandings of social inequality. However, social vision research has rarely prioritized the perspectives of marginalized group members. We offer a theoretical argument for diversifying understandings of social perceptual processes by centering marginalized perspectives. We examine (a) how social vision researchers frame their research questions and who these framings prioritize and (b) how perceptual processes (person perception; people perception; perception of social objects) are linked to group membership and thus comprehensively understanding these processes necessitates attention to marginalized perceivers. We discuss how social vision research translates into theoretical advances and to action for reducing negative intergroup consequences (e.g., prejudice). The purpose of this article is to delineate how prioritizing marginalized perspectives in social vision research could develop novel questions, bridge theoretical gaps, and elevate social vision’s translational impact to improve outcomes for marginalized groups.

**Public Abstract:**

Social vision research is a subfield of psychology and vision science which examines how people visually perceive social stimuli and what the downstream consequences of these perceptions are. Social vision work includes, for example, examination of how White people visually perceive racial minorities and how these perceptions lead to social categorizations of racial minorities as outgroups, and therefore contribute to behaviors such as stereotyping and prejudice. Social vision research has rarely prioritized the perspectives of marginalized group members. It therefore cannot fully explain the contributions of perception to intergroup relations, which are necessarily bidirectional. We offer a theoretical argument for diversifying understandings of social perceptual processes by centering marginalized perspectives to understand how people with marginalized identities see their social worlds. We believe that prioritizing these marginalized perspectives has the potential to contribute to the development of a psychological science with heightened capacity to improve the well-being of people with marginalized identities.

The notion of social vision was first introduced (see Adams et al., 2010) with the assumption that deriving social meaning from the visual cues of others is a biological imperative (i.e., necessary for our survival). The *Social Brain Hypothesis* (Dunbar, 1998) offered early evidence to support this claim. In this work, Dunbar reported a positive association between nonhuman primate species’ average neocortical volume—the part of the brain needed to reason about others—and the size of their social networks. This finding was hailed as evidence that the evolution of the human brain was in large part socially driven, with humans possessing both the largest brains and largest social networks of all primates. Albohn and Adams (2022) recently extended this work to faces as a way to underscore the role that social vision likely played in the evolution of the social brain, using similar techniques as Dunbar (1998). In addition to replicating Dunbar’s original findings, they found that primates’ capacity for facial expression is associated with both brain size and size of social networks, supporting their *Social Face Hypothesis*. Taken together, these findings suggest that vision serves a critical social function, one that conferred a survival advantage upon us. This perspective on social vision continues to shape emerging literature at the intersection of vision science and psychology.

The contemporary field of social vision often seeks to answer questions about how humans visually perceive social stimuli and how these perceptions translate into social categorization, activation of prior knowledge, and their downstream consequences (e.g., stereotypes; Adams et al., 2010). Historically, much of this literature focuses on face and, to a lesser extent, body perception; more recently, this work has expanded to include people perception (i.e., perception of groups; Alt & Phillips, 2021; Phillips et al., 2014, 2018). Social vision research advances our understanding of social perception in novel ways, particularly by focusing in the long-obscured visual antecedents of social phenomena; bringing theoretical insights from vision science to bear on a variety of social phenomena of prominent interest in psychology (e.g., stereotyping and intergroup relations) has fruitfully explicated the dynamic interplay of bottom-up visual processing (e.g., facial features and body size) and top-down cognitive processes (e.g., stereotypes and attitudes; Alt, 2019; Freeman & Ambady, 2011). Herein, we consider all research examining perception of social stimuli (i.e., faces and bodies) as social vision research, whether it identifies itself with that label or not.

Social vision research reveals new insights about how characteristics that observers bring to the task of social perception dynamically interact with objective stimulus parameters to inform social evaluations (Balcetis et al., 2016; Johnson et al., 2015). The notion that visual percepts are influenced by high-level cognitions contrasts with earlier views of visual percepts as unadulterated representations of the real world (see Johnson et al., 2015). However, developments in social vision (see Adams et al., 2010) allow us to understand the ways in which individual factors (e.g., identity and motivation) shape our perceptual attunements to, and thus conscious perceptions and memories of, physical aspects of social stimuli. To the extent that individuals bring differing characteristics, including social identities, motivations, and attitudes, to bear on perceptual inputs, these individuals will perceive objectively identical stimuli differently (see Balcetis & Dunning, 2006; Dunning & Balcetis, 2013; Xiao et al., 2016).

Our goal in this review of the social vision literature is to harness these insights and apply them to intergroup contexts, with a specific emphasis on the important ways in which marginalized social identities shape perceptual processes. We believe that the translational potential of social vision research—particularly with regard to improving outcomes (e.g., health disparities) among marginalized group members—has yet to be fully harnessed. This is largely due to a lack of focus on the perspectives of those who experience the brunt of the consequences of social categorization. Specifically, social vision research to date tends not to center those who are categorized as outgroups, who are marginalized in visual representations, and who are excluded and harmed by perceptually mediated prejudices and prejudice-mediated perceptions.

We argue that the translational potential of social vision research could be enhanced by prioritizing marginalized group perspectives. To this end, we provide readers with a background in the social vision literature and in the feminist theory upon which we base our arguments. We then critically examine (a) how social vision researchers frame their research questions and who is prioritized by these framings and (b) how perceptual processes (person perception, people perception, and perception of additional social informational stimuli we call *social objects*) are linked to group membership and thus comprehensively understanding these processes necessitates greater attention to marginalized perceivers. We conclude with a discussion of the theoretical and applied implications of prioritizing marginalized perceivers in social vision research and provide specific recommendations for researchers both within and outside of the social vision space. These recommendations outline ways to effectively prioritize marginalized perspectives and translate those perspectives into a better psychological science and better outcomes for marginalized group members. We also provide brief recommendations for editors and reviewers to manage research on marginalized perspectives.

## Author Positionality Statement

We draw upon our interdisciplinary backgrounds in social psychology, vision science, and women’s, gender, and sexuality studies to offer a feminist critique of the social vision literature and to develop a theoretical argument for diversifying understandings of social perceptual processes by transforming paradigms to center marginalized perspectives. The first author’s lived experiences with marginalized gender and sexual identities, in addition to identities marginalized within academia as an international and first-generation student, inform their standpoint as it is supported by their background in feminist psychology. The second author brings expertise in, and a formative and ongoing role in the development of social vision as an important area of inquiry to bear on these perspectives. Together, we develop our critique of representation in the social vision literature. The purpose of this article is to delineate how prioritizing marginalized perspectives in social vision research could pose and answer novel questions, bridge theoretical gaps, and ultimately elevate the translational impact of social vision research to improve outcomes for marginalized groups.

## A Feminist Social Vision

We propose that both social vision researchers who typically examine intergroup relations via dominant group perspectives, and psychologists who study marginalization/marginalized group perspectives but are not familiar with social vision theories or methodologies, would benefit from interdisciplinary enmeshment toward improving the translational impact of psychological science. We contend that this is particularly the case as it pertains to improving the well-being of marginalized group members. In line with our goal of writing to an interdisciplinary audience, we provide definitions and examples of key social vision concepts in Table 1.

**Table 1.** Key Terminology/Concepts and Citations.

Term/concept	Definition	Example	Canonical citations	Exemplary citations
Social vision	A subfield of psychology and vision science which seeks to answer questions about how humans visually perceive social stimuli and how these perceptions translate into social categorization, activation of prior knowledge, and their downstream consequences	Both the arguably innate capacity we have to read one another’s mental and emotional states from nonverbal behavior, and the top–down influence of expectations, motivation, personal history, and identity in shaping our visual perception.	Adams et al. (2010) Albohn & Adams (2016) Balcetis & Lassiter (2010) Johnson & Adams (2013)	Bagnis et al. (2019) Johnson et al. (2015) Shropshire & Johnson (2021) Xiao et al. (2016)
Person perception	The visual and cognitive processes we use to form impressions of other people, which are impacted both by the visual appearance and expression of a target and by the personal experiences and identities of an observer.	The impact of facial attraction, babyishness, and emotional expressions, as well as race and gender stereotypes on impressions such as perceived dominance and trustworthiness of others.	Caruso et al. (2009) Johnson et al. (2012) Macrae & Bodenhausen (2000)	Bogart & Matsumoto (2010) Friedman (2012, 2016) Hinzman et al. (2022) Kunstman et al. (2016) Lloyd et al. (2017)
People perception	The visual and cognitive processes we use to form impressions of groups of people	How we read a room, quickly assessing a group for their likely threat or benevolence toward us.	Haberman & Whitney (2009) Phillips et al. (2014)	Alt et al. (2019) Goodale et al. (2018)
Social object perception	The visual and cognitive processes through which we perceive objects and attribute to them a social meaning	Safety or threat cues, such as rainbow or confederate flags that can shape our perception of the environment.	—	Driskell & Trawalter (2021) Kruk & Matsick (2021) Vartanian et al. (2021)
Affordances	Opportunities to act on or be acted upon by a visual stimulus	A glass of water conferring upon us the opportunity to drink it.	Gibson (1979/2015) Zebrowitz et al. (2010)	Meagher (2020)
Attunements	A perceiver’s sensitivity to stimulus features associated with such affordances	A glass of water seemingly conveying “drink me” to one person, but screaming it at another person who just finished running a race.	Gibson (1979/2015)	Meagher (2020)

*Note*. Terms appear in order of scope and importance to the manuscript, with the broadest conceptual level appearing first and more narrow terms appearing later. Canonical citations represent overviews of the term/concept or seminal works in the area. Exemplary citations represent works which, while sometimes disparate in approach or scope, reflect the goals and sentiment of the current review and can serve as foundational to more inclusive social vision research. The social object perception row does not include canonical citations as we propose this category in the current work.

We argue that social vision offers many novel and creative methodologies capable of elucidating and representing marginalized perspectives. Without the consideration to prioritize these perspectives, the radical potential of these methods will remain constrained. In the following sections, we outline evidence for the role of perceiver characteristics in social vision, examine how social vision methods have been applied to the study of social inequality, and theorize a feminist approach to prioritizing marginalized perspectives in social vision, before turning to specific recommendations for framing research questions and conducting social vision research.

### The Role of Perceiver Characteristics in Social Vision

Adams and colleagues have adopted a functional approach to the study of social vision (e.g., Adams et al., 2010, 2017b; Weisbuch & Adams, 2012), drawing heavily on existing ecological models of vision and social perception (Gibson, 1979/2015; Zebrowitz, 1997). The ecological approach to visual perception assumes an interplay between the perceiver and the perceived, with emphasis on behavioral *affordances* and perceiver *attunements* (Gibson, 1979/2015). Affordances are defined as opportunities to act on or be acted upon by a visual stimulus. Put differently, the act of seeing a glass of water, for example, is directly linked to the functional behavior it confers upon us (i.e., “action vision”; Adams et al., 2017a); in this way, seeing a glass of water functionally says “drink me.” In turn, Gibson defined attunements as a perceiver’s sensitivity to stimulus features associated with such affordances. In this way, if someone finds themselves lost in a desert, that same glass of water might *scream* “drink me!” Critically, these behavioral affordances and perceptual attunements are often social in nature (Zebrowitz et al., 2010), shaped by learned expectations, individual differences, situational contexts, cultural influences, and so on.

It is important to note here that due, in part, to work in this domain, including examples highlighted in this review, there has been a resurgence of debate in the realm of perceptual psychology and philosophy (see Adams & Kveraga, 2015) regarding whether top-down influences such as motivation and expectations can infiltrate low-level perception. Firestone and Scholl (2016) landed the strongest attack on the notion of this sort of “cognitive penetrability” in the visual system, arguing that low-level visual processes are entirely encapsulated. Those who oppose the notion of cognitive penetrability nonetheless tend to agree that cognitive expectations can shape what we see, but they argue this occurs either at the “attentional selection stage prior to the operation of early vision, or in the perceptual selection or decision stage after the operation of early vision” (Pylyshyn, 1999, p. 414). Recently, others have argued that attention itself is a cognitive process that directly influences what we perceive (Lupyan, 2015). Notably, the ecological approach to vision highlights a primary role of attentional attunements in how we visually engage with the world around us, which is not in conflict with either side of this debate.

All said, this article is concerned with the impact of our conscious visual experiences of the world and their downstream impact on social attention, memory, and action, and thus we intend to make no philosophical claims regarding low-level perceptual penetrability. For extended views on this debate, one can refer to commentaries written in response to the Firestone and Scholl article, highlighting influences ranging from social, affective, and language, as well as detailing important neuroscientific considerations (e.g., O’Callaghan et al., 2016, 2017). What we believe is important to highlight here is that there is no debate regarding the notion that top-down influences (e.g., expectations, prior knowledge, etc.) have the potential to influence our visual experiences of the world.

Relevant to this debate, many unanswered questions remain about exactly how top-down influences, and particularly social identities, affect perceptual processes. Freeman and Ambady (2011) proposed a dynamic interactive theory of person construal attempting to bridge these gaps. They theorized that the perception of other people is accomplished through a dynamical interplay between higher-level social cognition influences (e.g., prejudice and motivational states) and lower-level sensory perceptual processes such that “perceptions of other people gradually emerge through ongoing cycles of interaction between social categories, stereotypes, high-level cognitive states, and the low-level processing of facial, vocal, and bodily cues” (p. 23). This model is suggested to be stimulated by continuous top-down input—a mode of stimulation particularly relevant to marginalized perceivers, who are likely to be continually aware of their marginalized identities and hypervigilant to cues of identity-based threat (see Pinel, 1999; Steele et al., 2002). However, the role of marginalized identities in shaping social perception has yet to be elucidated, despite the field’s attention to social inequality.

### Applying Social Vision to Social Inequality

Understanding the perceptual processes that inform social categorization in its initial stages allows for better understanding of the downstream social evaluation processes—such as stereotyping, prejudice, and discrimination—which are the focus of much literature in contemporary social psychology, social cognition, and fields beyond psychology concerned with topics pertaining to intergroup relations (e.g., sociology and women’s studies). Social vision research is thus well-positioned to make significant contributions to understanding social inequality and progressing toward improving the well-being of marginalized group members (see Bagnis et al., 2019; Johnson et al., 2015; Shropshire & Johnson, 2021; Xiao et al., 2016).

There is an awareness in the field that top-down influences, including social identities, shape social perception (see also Xie et al., 2021), and social vision research has historically focused on relevant processes of social categorization and the downstream, intergroup dynamics thereof (e.g., stereotyping, prejudice). Nonetheless, social vision research has rarely prioritized the perspectives of marginalized group members. Work in social vision is instead often agnostic to perceiver identities (Xie et al., 2021) or otherwise predominantly focuses on dominant group members’ perceptions of marginalized targets (e.g., sexual minorities, racial minorities, and gender minorities) to the near exclusion of marginalized perceivers (see also Willadsen-Jensen & Ito, 2008). Work oriented toward dominant group members’ perceptions of marginalized group members has been important in elucidating visual mechanisms underlying dominant group members’ prejudiced attitudes toward minorities (e.g., processing disfluency, Lick & Johnson, 2013, 2015) and how prejudices can be mediated by perceptual processes (see Shropshire & Johnson, 2021).

One prominent example of this phenomenon demonstrated by Caruso et al. (2009) is that political partisanship influences perceptions of the skin tone of biracial political candidates; to the extent that people’s partisanship matches with a biracial candidate, they are likely to perceive that candidate’s skin tone as lighter. Caruso and colleagues demonstrated that for then-presidential candidate Obama, the extent to which people rated artificially lightened photographs of him as representative of his actual skin tone was related to their voting intentions and behavior such that those whose partisanship was aligned with Obama’s perceived him as having a lighter complexion and were more likely to vote for him than were those who did not match with his partisanship and perceived him as having a darker complexion. This work demonstrates that “partisans not only “darken” those with whom they disagree, but also “lighten” those with whom they agree” (p. 20170). Further research demonstrates that stereotypical beliefs about social groups influence how traits and emotions are attributed to faces and bodies of individuals belonging to those groups (e.g., Hugenberg & Bodenhausen, 2003, 2004; Inzlicht et al., 2008; Wilson et al., 2017; see also Freeman et al., 2010); for example, Hugenberg and Bodenhausen (2003) demonstrated that, among White participants, implicit racial prejudice was associated with increased likelihood of perceiving anger in Black faces, but not White faces. Racial (and other) biases influence initial social perceptions, and these categorizations have important behavioral consequences for intergroup relations and downstream outcomes of marginalized group members (e.g., Eberhardt et al., 2006; Lick & Johnson, 2015; see also Freeman et al., 2010).

This line of research, however, has largely failed to interrogate the social vision of marginalized group members. In part, this may be due to a focus on intergroup relations broadly construed which tends to ignore the broader context of intergroup power relations; that is, much work in the domain of social identity effects on perception minimizes the effects of the broader hierarchical social contexts in which social identities and group memberships are embedded, viewing ingroup social identities as a starting point rather than an effect of these dominance hierarchies (Kteily & Richeson, 2016). Orienting toward these social hierarchies by acknowledging the unique perspectives of those in different social locations (dominant versus marginalized group members) is a promising avenue for the expansion of social vision.

### Prioritizing Marginalized Perspectives

We argue that beyond their roles as targets in experimental paradigms, the perspectives of marginalized group members add significant value to the study of intergroup relations (Matsick et al., 2021, 2022; Shelton, 2000), the bidirectional nature of which is obscured by the near-uniform focus on dominant group perspectives in psychological research (Roberts et al., 2020; Shelton, 2000; see also Matsick et al., 2022). The absence of these marginalized perspectives in much of the psychological literature, particularly that pertaining to intergroup relations, “presents a serious challenge to understanding intergroup dynamics and concomitant disparities” (Matsick et al., 2022, p. 43). Including marginalized perspectives in this literature would enhance the relevance of psychological science to broader audiences as well as the accuracy and generalizability of psychological research (Cole, 2009; C. Fine, 2018; M. Fine & Gordon, 1989; Hinzman et al., 2022; Sprague, 2005). Social psychology in particular has begun to make inroads into understanding marginalized perspectives, particularly with regard to marginalized group members’ experiences of stigmatization. For example, Major, Gramzow, et al. (2002) examined marginalized group members’ perceptions of personal discrimination, and Shelton’s (2000) call to focus on Black people’s experiences in intergroup contexts birthed a number of studies in this vein. Furthermore, emerging literature on environmental cues to threat and safety tends to focus on marginalized people’s perceptions of these cues (see Kruk & Matsick, 2021). However, this approach of prioritizing marginalized perspectives is rarely incorporated into social vision research.

To begin to remedy this, we draw upon feminist theorizing to elucidate the value of prioritizing marginalized groups’ perspectives. In particular, feminist standpoint theory (Haraway, 1988; Harding, 2004) offers an account of socially situated knowledge, such that all understandings of the world around us are shaped by our unique social locations and identities. Individuals with differing identities, then, are privy to different perspectives and understandings; individuals with differing identities encounter and perceive their worlds differently, even if objective parameters are uniform. Linking social locations to perception, Harding (1992) notes that “observers do change the world that they observe” (p. 463), and Haraway (1988) “insist[s] on the embodied nature of all vision” (p. 581). These feminist views are theoretically compatible with early influences in social cognition such as the “New Look” perspective on vision, which posited that perception is subject to top-down influences such as motivational factors (Bruner & Goodman, 1947; see also Balcetis & Dunning, 2006). This “New Look” perspective is embodied in Bruner and Goodman’s finding that economically disadvantaged children perceived coins as larger than identically sized but nonvaluable objects, while children from well-off families did not demonstrate this perceptual bias.

Extending from the notion that perception differs by social location, among other top-down influences (see also Balcetis & Dunning, 2006; Xiao et al., 2016), we argue, in line with feminist standpoint theorizing that the unique perspectives of marginalized group members offer valuable insight into intergroup relations and dynamics, and that this knowledge is inaccessible to dominant group members (Haraway, 1988; Harding, 2004; Rolin, 2009; see also Matsick et al., 2021). An accurate account of perceptual contributions to intergroup relations—that is, an accurate account of social vision—necessitates diversifying understandings of social perceptual processes by centering marginalized perspectives.

However, we caution that it is not enough to simply add marginalized perspectives into existing paradigms. We return to this point in our discussion of *Framing Social Vision*, but here we note that the simple inclusion of marginalized group members, particularly as a comparison group to dominant group members, has the potential to reinforce stereotypical and discriminatory conceptualizations of marginalized groups as deviating from dominant norms (e.g., Cole, 2009; McClelland & Dutcher, 2016). We thus argue that, to develop a truly inclusive social vision, broader paradigm shifts are required. We offer suggestions, examples, and notes of caution on these paradigm shifts in the following.

### Framing Social Vision

In line with Harding (1992), we believe that “starting off thought from [marginalized] lives provides fresh and more critical questions about how the social order works than does starting off thought from the unexamined lives of members of dominant groups” (p. 451). In an effort to elucidate the “fresh” potential of a social vision that prioritizes marginalized perspectives, we begin by addressing the types of questions that social vision research asks, and has the potential to answer.

Social vision research largely centers on questions regarding “perceptions of the most important class of objects in our perceptual environment—other people” (Johnson & Adams, 2013, p. 634). The social vision literature, distinct from either the literature on vision or on social psychology more broadly, aims to link these perceptual processes to social phenomena. In doing so, much of this literature explicates the perceptual bases of stereotyping, bias, prejudice, and stigmatization, providing a novel lens on intergroup dynamics. In sum, this literature examines *how* people perceive social stimuli, and the social outcomes of these processes; we suggest that there is need for more focus on *who* is perceiving social stimuli.

In the domain of social stereotyping work, social vision research tends to include marginalized group members only as the targets of perception, and not as perceivers themselves (Hinzman et al., 2022; see also Major & O’Brien, 2005). Yet, the perceptual processes underlying findings from these paradigms are often assumed generalizable (Hinzman et al., 2022), obscuring how perceptual processes among marginalized group members—particularly in the context of intergroup perception—may differ from those among dominant group members. As Hinzman et al. (2022) note in their re-examination of cross-race face processing, ultimately, “. . . few models or studies explore the extent to which a perceiver’s group identity, particularly stigmatized identity, influences face perception processes” (Hinzman et al., 2022, p. 2). In line with Hinzman and colleagues (2022), we argue that theoretical and methodological approaches to social vision research broadly defined must do more to incorporate marginalized group perspectives. We believe that an initial step in this direction would be to reconceptualize how social vision researchers ask their research questions.

Notably, we argue against the mere incorporation of marginalized perspectives into existing paradigms for conducting social vision research. For example, one might seek to include marginalized perspectives by adapting a paradigm to examine the inverse relation; a study once examining how heterosexual people detect cues to bisexuality in faces might be reversed to examine how bisexual people detect heterosexuality from faces. Such a reversal fails to account for the unequal distribution of power between groups, and thus may render itself irrelevant. Knowing how heterosexual people detect facial cues to bisexuality is relevant because such perceptions are an antecedent of bisexual prejudice (Lick et al., 2015), but the contribution of the inverse is unclear; that is, it is not clear whether the perception of heterosexuality meaningfully signals anything to bisexual people. One may reason that facial cues to heterosexuality signal a threat of prejudice, given heterosexual people are typically the perpetrators of heterosexism. However, bisexual people face prejudice from both heterosexual and queer people (Matsick & Rubin, 2018); therefore, facial signals to heterosexuality specifically are not likely to be a useful or meaningful signal to bisexual people at least with regard to experiences of prejudice and who they are likely to encounter prejudice from. More meaningful visual signals for bisexual people might include cues to belonging or safety from prejudice, such as bisexual flags or people with androgynous gender expressions (see Hartman, 2013). To understand bisexual people’s visual experiences, research must therefore organize around bisexual people’s perspectives rather than simply reversing paradigms to treat former targets as perceivers and vice versa.

Many social vision researchers orient their work about marginalized group members in this way—prioritizing dominant group members as perceivers and marginalized group members as targets—in pursuit of contributing to valuable goals including the reduction of bias, prejudice, stigmatization, and inequality (see Freeman et al., 2016; Johnson et al., 2015; Shropshire & Johnson, 2021; Xiao et al., 2016). Often, these framings suggest that if we could understand the perceptual roots of dominant groups’ biases toward marginalized groups, we could intervene at this early stage and thus reduce intergroup conflict and disparities (e.g., Shropshire & Johnson, 2021; Xiao et al., 2016). Such paradigms thus focus on dominant group members as perceivers, and marginalized group members as targets, in an effort to intervene upon these perceptual pathways to bias. We believe this reasoning—that prioritizing dominant group perspectives provides an intervention target suited to improve the well-being of marginalized group members—may, paradoxically, be why many social vision researchers opt not to skew their paradigms toward marginalized perspectives.

Critically, we do not believe this is justification enough. Indeed, a large body of feminist psychological literature backs this point—that mere inclusion of marginalized populations “will not automate a radical shift in science because it does very little to promote the paradigm shifts required for building an inclusive and intersectional psychology” (Matsick et al., 2021, p. 418; for review, see McCormick-Huhn et al., 2019). Indeed, feminist scientists reject what is often called the “add women and stir” approach, which describes the approach of adding women—or any marginalized group—to an existing framework without changing that framework to be inclusive to women, instead leaving these frameworks structured by the interests and understandings of dominant group members (Harding, 1995; Matsick et al., 2021). Such minor tweaks, as opposed to radical restructuring of paradigms, will always disproportionately benefit the most privileged individuals within marginalized communities (e.g., cisgender, White, middle-class gay men; Guyan, 2022; see also Siegel, 1997).

For example, simply reversing paradigms as in the example above—to examine how bisexual people detect facial cues to heterosexuality—represents a minor tweak whereby the participant/target relationship is reversed, but contextual factors are not considered. A radical restructuring of paradigms would necessitate advocating for bisexual people’s positionality, understanding what visual cues are relevant to them and their social experiences, and designing studies addressing perception of these cues. To achieve these ends, researchers may have to consider unique methodologies for understanding bisexual people’s perspectives, including adopting qualitative methodologies or community-based approaches. We discuss opportunities to include marginalized perspectives via the use of these methodologies later in the article, and provide explicit recommendations for researchers interested in prioritizing marginalized perceivers in their work. Notably, however, we do not advocate for wholesale exclusion of dominant group members as perceivers in social vision research. Some phenomena of interest among dominant group members may not generalize to marginalized groups (and vice versa), given the inequitable distribution of power and resources between these groups.

To meaningfully include diverse marginalized perspectives in social vision research, we must consider the unequal distribution of power in intergroup relations and contextualize marginalized perspectives in ways that highlight the value of these perspectives (see Cole, 2009; McCormick-Huhn et al., 2019). In the following section, we seek to provide this consideration and contextualization. Drawing upon theorizing on intergroup relations, stigma, vision, and perception, we elucidate how the marginalized identities of perceivers—not just targets—might contribute to social vision processes, and attempt to move social vision beyond questions about the perception of social identities to questions about how identities influence perception.

### The View from Below: Marginalized Group Perspectives

We delineate theoretical arguments to support the idea that centering marginalized perspectives would allow for a more comprehensive social vision literature with a greater capacity to improve marginalized group members’ well-being. We explore how marginalized social identities may shape perceptual processes at three levels: *person perception* (the area that constitutes the bulk of social vision research, examining perception of individual faces and bodies); *people perception* (an emerging area of research in social vision which examines perception of groups); and perception of *social objects* (a novel class of social stimuli we argue has relevance to social vision which has been obscured by the predominant focus on dominant perspectives). In addition, we consider how to integrate intersectional perspectives with these theoretical contributions. Within each subsection, we present existing social vision work, highlight how prioritizing marginalized perspectives could help advance this work, and illuminate future directions building from contemporary social vision work which would have increased potential for translational impact (see Table 2 for a summary of future directions and recommendations for social vision research).

**Table 2.** Recommendations for Prioritizing Marginalized Perspectives in Three Areas of Social Vision Research.

Theme	Recommendations	Examples of application	Relevant literature
Person perception	*Attend to experiential and cognitive factors.* Consider how experiences of discrimination may shape vigilance and attentional resources relevant to person perception processes.	Review literature on minority stress and other relevant psychosocial moderators in relation to implications for attention and cognitive resources; prioritize marginalized voices in understanding experiences of discrimination	Hinzman et al. (2022) Major, Quinton, & McCoy (2002) Meyer (1995) Matsick et al. (2022)
	*Orient research designs around marginalized perceivers*. Adopt designs that prioritize dyadic interactions of interest to marginalized group members.	Consider social issues relevant to marginalized groups and how person perception contributes to these issues; consider assessing both similarities and differences between marginalized and dominant group members	Haraway (1988) Harding (2004) Matsick & Conley (2016) Matsick et al. (2021)
	*Consider how intersectional forces shape perception.* Attend to the multifarious nature of perceiver identities.	Reflect on and report both marginalized and privileged identities that perceivers may hold; collect comprehensive demographic information; look to feminist literature on best practices for intersectional research	APA (2019) Cole (2009) Buchanan et al. (2021) Else-Quest & Hyde (2016a, 2016b) Guyan (2022)
People perception	*Attend to the importance of group characteristics to marginalized perceivers*. Critically examine how group characteristics such as diversity hold particular importance for marginalized perceivers.	Review literature on diversity as a safety cue, and in relation to social identity threat more broadly; consider marginalization in context; consider who is represented and who is left out	Alt et al. (2019) Emerson & Murphy (2014) Goodale et al. (2018) Hall et al. (2018) Stewart (1998)
	*Model theories of group perception which acknowledge and center marginalized perceivers*. Examine potential heterogeneity both between dominant and marginalized perceivers and within marginalized perceivers in people processing.	Critically examine how marginalization may shape people perception at all stages of people perception; test models with both marginalized and dominant perceivers to assess generalizability; look for both similarities and differences in processing strategies	Alt & Phillips (2021) Major et al. (2013) Phillips et al. (2014)
Social object perception	*Consider how stimuli may hold different meaning to marginalized perceivers*. Identify assumptions about which stimuli convey social meaning.	Reflect on assumptions embodied in the literature; prioritize marginalized voices in understanding perceptual experiences; consider how vigilance shapes perception of a variety of stimuli	Kruk & Matsick (2021) Murphy et al. (2007) Murphy & Taylor (2012) Oswald et al. (2022)

*Note.* Themes are organized by order of appearance in the article. The relevant literature column is intended to point readers to work which provides background information on relevant theory, constructs, and/or measurement tools.

#### Person perception by marginalized group members

The perception of individual persons has been the predominant focus of research in social vision, particularly the perception of faces and, to a lesser extent, bodies. As reviewed above, this research, when it seeks to understand intergroup phenomena, has predominantly focused on target group membership (i.e., how people, usually dominant group members, and perceive marginalized targets) rather than on perceiver group membership and its influences on perception (see also Hinzman et al., 2022; Xie et al., 2021). However, there is reason to expect that perceiver group membership, particularly among marginalized perceivers, influences perceptual processes.

For example, a large body of social psychological literature elucidates the psychological consequences of exposure to chronic stigmatization and discrimination (see Link & Phelan, 2001; Major & O’Brien, 2005; Matsick et al., 2020). These experiences of discrimination leave marginalized individuals (hyper)vigilant to social identity threat, or concern about how they will be treated by others based on their marginalized identity (Steele et al., 2002). This hypervigilance, along with unequal distributions of power and control of resources, may compel marginalized group members to pay greater attention to dominant group members than vice versa (Dunham et al., 2014; Link & Phelan, 2001). As noted by Hinzman and colleagues (2022), these experiential and cognitive factors are also implicated in face perception processes (see also Young et al., 2012), suggesting that perceiver’s experiences of marginalization are likely to influence social vision. Theorizing these influences in the context of cross-race face processing and memory, Hinzman et al. (2022) propose that marginalized group members’ heightened exposure to dominant outgroup members (relative to ingroup members), heightened motivation to attend to dominant group members, given their disproportionate power and strained cognitive resources—resulting from experiences of discrimination—in interactions with dominant group members and dominant contexts all contribute to unique perceptual processes among marginalized group members. Hinzman et al. also issue a call for greater attention to marginalized perceivers specifically in the context of face processing and memory.

We extend that call to all social vision research focused on intergroup perception, and argue that not only should future research “include stigmatized minority perceivers into their research designs” (Hinzman et al., 2022, p. 11), but that social vision research should orient its designs around these perceivers. As one example of research oriented toward a marginalized perspective while utilizing traditional social vision methodologies, we examine Lloyd et al.’s (2020) examination of race-based differences in mental representations of police. In this study, Lloyd and colleagues used reverse correlation—a data-driven procedure for estimating participants’ mental representations (Mangini & Biederman, 2004; see Dotsch & Todorov, 2012 for review)—to assess differences in Black and White civilians’ perceptions of police. Reverse correlation procedures use a series of base images with overlaid noise, which participants rate in a serial task, to estimate composite mental representations, called “classification images,” of a given trait or identity.

Lloyd et al.’s work, published during the proliferation of the Black Lives Matter movement, which focuses on Black people’s liberation from systemic discrimination and injustice, particularly at the hands of the legal system (Black Lives Matter, n.d.), prioritizes Black participant’s perspectives to understand a social issue relevant to these participants, and particularly to their marginalized racial identity. Lloyd et al. (2020) demonstrate that Black Americans have more-negative, less-positive, and more-dominant mental representations of police than do White Americans. This work thus extends what we know about attitudes toward police—also an important area of inquiry for individuals marginalized by policing systems—to the perceptual realm. Lloyd and colleagues describe some potential implications of the work with regard to interactions between civilians and police, and how these may unfold differently depending upon their perceptual starting point. This work prioritizes marginalized perspectives to understand a perceptual process relevant to marginalized group members, and elucidates differences between dominant and marginalized group members’ perceptions of individuals belonging to a specific group (i.e., police). The work is structured around the notion that Black people’s experiences with police differ from those of White people, and is thus oriented to that marginalized standpoint.

We believe that this work represents a step forward in social vision research with regard to representing marginalized perspectives to understand intergroup dynamics. However, we also note that there are potential limitations when using a group differences approach (i.e., comparing dominant to marginalized group members); searching only for group differences, particularly when not theoretically motivated, “may essentialize social groups and reproduce discriminatory science” (Matsick et al., 2021; see also Wyer, 2018). Examinations of group differences should thus be theoretically supported. An alternative approach is to examine group similarities (see Cole, 2009; Matsick et al., 2021; Syed & Kathawalla, 2021), which can inform understandings of where groups overlap in perceptual strategies. Another approach to center marginalized groups is to specifically orient work toward their perspectives and experiences, and prioritize the inclusion of marginalized perceivers as participants. Dominant group members need not be included in all paradigms, and it may not make sense to include dominant group members in paradigms centered on marginalized perspectives (much as it may not make sense to include marginalized group members in paradigms centered on dominant perspectives, which we suggest may underlie their lacking representation in existing social vision research).

Furthermore, there may be significant value in examining differences *within* marginalized groups. Historically, little attention has been paid to within-group differences among marginalized group members, though there are important exceptions in the literature (see Major et al., 2016). Focusing on individual differences among marginalized group members can elucidate variability in their perceptions and experiences of prejudice; for example, individual differences in stigma consciousness (Pinel, 1999) partially explain differences among marginalized group members in health outcomes (e.g., mental and physical health outcomes among sexual minorities; see Figueroa & Zoccola, 2015). Social vision research examining the broader construct of *suspicion of White’s motives*—the belief that positive overtures from White people are motivated more by social concerns about appearing prejudiced than by personal commitments to egalitarianism (Kunstman et al., 2016; Major et al., 2013, 2016)—taps into the significance of within-group variability among marginalized perceivers. Specifically, social vision studies on suspicion of White’s motives find that individual variability in suspicion of White’s motives among Black perceivers predicts differential perceptions of how trustworthy and authentic White people are, and how threatening White people’s smiles are (Kunstman et al., 2016; Lloyd et al., 2017). This exemplary social vision work demonstrates the importance both of examining marginalized perceivers and of examining heterogeneity among these perceivers.

##### Intersectional considerations

The approaches we have detailed thus far to developing a social vision centering marginalized perspectives have primarily been discussed in terms of perceivers’ singular marginalized identities (e.g., race or gender). However, as social vision research has long acknowledged in the study of what are often called compound social cues (e.g., Adams et al., 2017b; Adams & Kveraga, 2015; Albohn & Adams, 2016; Goff et al., 2008; Kang & Bodenhausen, 2015), visual targets have multiple, intersecting social identities which influence how they are perceived. For example, social vision work demonstrates that race and gender together influence sex categorization (Johnson et al., 2012), gendered facial cues influence sexual orientation perception (Freeman et al., 2010), and social status cues influence race perception (Freeman et al., 2011). Social vision has the potential to adopt truly intersectional approaches to call to attention the “multidimensionality” of marginalized subjects’ experiences (Crenshaw, 1989, p. 139); to reject single-axis constructions of identity and instead forward a more complex notion of identity such that “subjectivity is constituted by mutually reinforcing vectors of race, gender, class, and sexuality” (Nash, 2008, p. 2). Acknowledging the multiple and interlocking identities of individuals, and perhaps more importantly, the ways in which privilege and marginalization interact to shape these identities (see Crenshaw, 1989, 1991; hooks, 1984), is necessary to truly understand how intergroup processes occur in reality.

Feminist psychologist Stephanie Shields delineates how “intersectionality frequently becomes redefined as a methodological challenge” in the social and behavioral sciences (Shields, 2008, p. 305). Available methodological tools impede the radical potential of intersectionality and often treat identities “much more like independent factors within a conventional factorial research design” (Shields, 2008, p. 304) than like mutually constituted axes. Treating intersectionality as an interaction term defies the fundamental nature of intersectionality, as this type of analysis assumes that the two (or more) factors contributing to the interaction are independent of one another. Cole (2009) and Else-Quest and Hyde (2016a, 2016b) provide excellent guidance on incorporating intersectional approaches into psychological research paradigms broadly.

We posit that social vision methodologies offer particular opportunities for incorporating intersectionality into psychological research. In particular, some bottom-up approaches such as the reverse correlation technique (e.g., Dotsch et al., 2008) allow for estimating participants’ mental representations of individuals with intersecting identities—that is, participants in a reverse correlation study could create classification images of targets with very specific, intersecting identities, the specificity of which would be very difficult to assess in more traditional stereotyping paradigms. This technique could allow social vision researchers to better understand how perceptions of individuals are shaped by multiple systems of power and oppression; for example, one could examine how perceivers’ endorsement of racism, sexism, and classism co-constitute representations of wealthy and poor Black women’s faces. Reverse correlation procedures have been harnessed to examine the interaction of compound social cues, such as the interaction of gender and emotion whereby masculine faces tend to be perceived as angry, and feminine faces tend to be perceived as happy (Brooks et al., 2018; though see Cone et al., 2021 for critiques of some approaches to reverse correlation).

We suggest also that the acknowledgment of the intersectional nature of social categories should extend to perceivers. Perceivers are shaped by intersecting systems of oppression, such that their perceptions are likely to be influenced by not only their membership in certain social groups (e.g., identifying as women) but more importantly by the systemic forces which reify these groups (e.g., patriarchy and sexism). Extending acknowledgment of intersectionality to perceivers may take multiple forms, including collecting more comprehensive demographic data from participants, acknowledging how multiple forms of oppression constitute participant and target identities and incorporating these into theorizing, and more explicitly acknowledging the limitations of samples which are marginalized only on a singular identity, or which homogenize diverse participants due to one shared identity. With regard to demographic collection and reporting, we suggest social vision researchers collect and report not only the traditional categories of age and gender but also gender status (cis or transgender), race/ethnicity, sexual orientation, social class, and any additional identities that are contextually relevant. Feminist and critically oriented psychologists provide many resources for inclusive best practices on collecting and reporting demographic data (e.g., Beischel et al., 2022; Guyan, 2022; see also American Psychological Association [APA], 2019). Collecting and reporting more comprehensive participant demographics allows for intersectional analyses at the level of the participant, and may assist in identifying subgroups with different (or similar) perspectives (see also Buchanan et al., 2021).

Defining identity from an intersectional perspective means being more specific about who we mean when we talk about a specific category of people, and about how systems of power and oppression, in relation to their identities, shape their perception. Employing an intersectional analysis defined this way allows for models that better explain the experiences of diverse individuals. For example, theorizing about gay men’s perceptions of some phenomena is often really theorizing about *young cisgender White* gay men’s perceptions; these perceptions may not be shared by, for example, Black gay men, elderly gay men, or people with any other combination of identities which also include being gay men. Being more explicit about the diversity of a sample can help either orient toward intersectional theorizing (e.g., contextualizing gay men’s experiences as they are also shaped by systemic racism, ageism, classism, etc.) or avoid further marginalizing individuals who are not represented in a sample by assuming that the sample’s experiences generalize to them (e.g., assuming that White gay men’s experiences generalize to Black gay men). However, as we note above, orienting toward differences between groups—for example, between White gay men and Black gay men—should be theoretically motivated; it should not be assumed that one group’s experiences (or one group member’s experiences) generalize to all members of that group, nor should it be broadly assumed that there are essential differences between all members of all groups (see also Cole, 2009). Intersectional forces should be considered as potential predictors of perceptual processes, but only insofar as these ideas are theoretically motivated and, perhaps more importantly, motivated by the experiences of individuals with the identities of interest.

Before researchers begin to study specific intersecting identities or intersectional experiences (see Jackson et al., 2021), they should consider whether their research questions are best served by a comparative approach or by an approach which focuses only on a single group. This choice is best informed by members of the group of interest; researchers should engage strategies from participatory action research to work with members of the group of interest to develop relevant research questions and study designs (see also Buchanan et al., 2021; Dupont, 2008; Lykes & Crosby, 2014). These strategies and approaches for prioritizing marginalized perspectives apply also to the processes of people perception and what we refer to as social object perception, which we discuss next.

#### People perception by marginalized group members

A developing literature on people perception, or the perception of groups of people, examines how person perception processes, which tend to focus on the perception of individuals, extend to perceiving the social properties of groups. Similar to the social vision tradition in person perception, recent work on people perception bridges decades of literature from vision science with work on social perception. In particular, much work on people perception has drawn on the vision science phenomenon of *ensemble coding*—the ability to extract summary statistical properties from sets of objects (e.g., the average size of a set of circles; Ariely, 2001; see also Phillips et al., 2014, 2018) While initial work on ensemble coding focused on low-level features (e.g., color, shape, size), more recent work indicates that this perceptual acuity also extends to higher-level, social stimuli such as emotions and identities (e.g., Haberman & Whitney, 2009; Phillips et al., 2014; see also Alt & Phillips, 2021). For example, people are adept at perceiving average race, gender, and dominance, as well as the variance of these properties, in groups of faces (Phillips et al., 2018).

People perception is particularly unique because groups have unique characteristics which can emerge only at the level of the group; two of these which have been most richly theorized are hierarchy and diversity (see Phillips et al., 2014). These characteristics can emerge only when multiple people are present, and thus have not been assessed in traditional social vision paradigms. Despite the apparent relevancy of perception of diversity to marginalized identities, people perception processes, much like person perception processes, have rarely been considered from the perspective of marginalized group members (though we discuss some exceptions below). The capacity of these processes to be harnessed in understanding intergroup relations, however, has been previously noted (Lamer et al., 2018).

Although to date, we are aware of little work that has examined social vision through the lens of marginalized identities, work in the cross-cultural domain has begun to shed light on how identity can shape visual experience. The socialized attention hypothesis (Park & Kitayama, 2011), for instance, predicts that differences in interdependent versus independent self-orientation styles will differentially influence holistic versus focused visual processing in East Asians versus Westerners, respectively. Consistent with this hypothesis, Westerners have been found to be biased to processing focal information (e.g., a single face ignoring surrounding context), whereas Easterners are more biased toward processing global information (e.g., the surrounding context, including other people) when perceiving individual faces (e.g., Blais et al., 2008; Caldara et al., 2010).

Such cultural differences are thought to be due to differences in spatial frequency attunements, such that Westerners tend to rely more on high spatial frequency information from foveal vision, whereas Easterners appear to rely more on extra-foveal vision (Miellet et al., 2013). This local versus global contrast difference emerges very early (e.g., under 80 ms) in visual perception (Kitayama & Murata, 2013; Lao et al., 2013), showing how tightly bound social influences in vision can be. When asked to report on the emotion of one face embedded in a crowd of other expressive faces, Easterners, more so than Westerners, integrate the emotional expressions of others into their judgments (Masuda et al., 2008). Finally, in a recent test of the social attention hypothesis examining the average emotion displayed from arrays of faces (i.e., “crowd” perception), Korean observers outperformed U.S. White observers (Im et al., 2017). In light of the overwhelming evidence of psychology’s WEIRDness, or overrepresentation of Western, Educated, Industrialized, Rich, and Democratic samples (Henrich et al., 2010; Rad et al., 2018), we believe this cross-cultural work, and its contributions to understanding how social identities shape perceptual processes, represent a window into the transformative potential of highlighting the perspectives of groups historically marginalized in the social vision literature and in the psychological literature more broadly.

We believe that people perception is particularly relevant to marginalized perceivers due to the link between visual representations of diversity and social identity threat. Social identity threat can be activated by environmental cues—often referred to as situational cues (Murphy et al., 2007; Murphy & Taylor, 2012)—which signal one’s belonging, or lack thereof (i.e., identity threat), in a particular space. For example, Murphy and colleagues (2007) demonstrated that women majoring in math, science, and engineering who viewed a video of an academic conference in their field experienced greater social identity threat when there was a significant gender imbalance portrayed in the video versus a balanced ratio of men to women depicted; women who viewed the unbalanced video reported lower belonging, less desire to participate in the conference, and heightened cognitive and physiological vigilance. Although this work did not focus on the visual aspect of group perception, the findings indicate the potential for a lack of minority representation to activate social identity threat. Conversely, representation of diversity can serve as a safety cue: an aspect of the environment or social setting that communicate one’s identity is valued and that the threat of discrimination is limited (Chaney et al., 2019; Kruk & Matsick, 2021). For example, having Black employees represented in company brochures leads Black participants to trust the institution more (Purdie-Vaughns et al., 2008).

These findings demonstrate that visual perception of diversity is linked to downstream social evaluations of groups among marginalized group members, yet social vision work on people perception has largely failed to examine these processes. However, there are some exceptions which include both dominant and marginalized perceivers and provide valuable insight. For example, in a study of ensemble coding of gender, Alt et al. (2019) found that as the number of male faces in an ensemble increased, men and women’s perceptions of the group’s sexism increased; notably, there did not appear to be major contributions of perceiver gender to these effects (i.e., few differences between men and women perceivers). Although participants were exposed to the ensembles of faces for only 500 ms, they were able to form downstream evaluative judgments of these ensembles based on their statistical properties (i.e., average gender). Goodale et al. (2018) used a similar ensemble coding paradigm, finding that both men and women anticipate increased belonging in groups as representation of members of their own gender increases. These authors provide that they do not believe their findings to be unique to women, and suggest that men who are contextually numerical minorities (e.g., men in fields such as nursing or education) may benefit from the identity safety cue of representations of gender parity. Similar findings have been indicated in other research focusing on safety cues targeted at men in contexts where men tend to be numeric minorities (e.g., prenatal doctor’s offices; Albuja et al., 2019), indicating the necessity of attending to context when considering who is marginalized.

We theorize that people perception processes are likely unique among marginalized group members in important ways. We draw upon Phillips et al.’s (2014) Selection, Extraction, and Application framework of people perception to elucidate our reasoning. The Selection, Extraction, and Application model proposes that (a) perceivers spontaneously *select* people into groups based on a number of bottom-up (e.g., target proximity, similarity) and top-down (e.g., attentional goals and existing knowledge) influences; (b) perceivers *extract* summary statistics (e.g., average and variance) from the groups formed in stage 1; and (c) perceivers form and *apply* judgments about the group based on these perceptual summaries. We believe that particularly at the extraction and application stages, marginalized group membership may influence people perception processes.

Specifically, during the selection stage, motivational goals, such as the desire to be inclusive or exclusive, are proposed to influence the selection of members into a perceptual group (Phillips et al., 2014); for example, motivation to be inclusive prompts heightened selection of racially ambiguous individuals into White people’s ingroups, and memory for ambiguous faces is improved when they are associated with the ingroup (Pauker et al., 2009). Marginalized group members may experience a heightened desire to be inclusive, not only of members of their own marginalized group but also those of other marginalized groups, relative to dominant group members. Key insights from intraminority intergroup relations inform our theorizing of this heightened inclusivity. For instance, marginalized group members may experience a “common ingroup identity” (Richeson & Craig, 2011); this identity would then extend across marginalized identities such that intraminority attitudes will arguably be positive, which could prompt desire for inclusivity. In addition, research from the safety cue literature indicates intraminority cue transfer, such that cues to the safety of one marginalized group (e.g., numerical representation of women) could also prompt safety for members of additional marginalized groups (e.g., Black men; see Kruk & Matsick, 2021). Thus, marginalized group members’ theorized heightened desire for inclusivity may prompt differential selection of individuals into groups, relative to selection by dominant group members.

During the application stage, Phillips et al. (2014) propose that relevant knowledge about groups of people shapes the weight of the perceptual summary (versus other knowledge) in contributing to judgments of the group. Marginalized group members pay more attention to and have more knowledge of dominant group members than vice versa (as a result of hypervigilance to threat; e.g., Dunham et al., 2014; Link & Phelan, 2001). They are also likely to have semantic knowledge of marginalized groups, particularly those they belong to. Therefore, it is possible that perceptual summaries may not be primary drivers of marginalized group members’ perceptions of groups. Perceptual summaries may contribute more to dominant group members’ perceptions of people, particularly when those people are comprised at least to some degree of marginalized group members. Conversely, the Selection, Extraction, and Application model (Phillips et al., 2014) proposes that motivational and cognitive resources are inversely related to the use of perceptual summaries in group judgment. Marginalized group members may have reduced motivational capacity and cognitive resources to attend to group judgment, given the cognitive load of minority stress brought on by chronic experiences of discrimination (see Link & Phelan, 2001; Major & O’Brien, 2005; Matsick et al., 2020); this would suggest that marginalized group members may rely more heavily on group percepts when applying judgments to groups of people.

However, it is also possible that marginalized group members dedicate significant motivational and attentional resources to forming group impressions, given the uniquely heightened implications of these perceptions for marginalized group members (e.g., misperceiving the inclusivity of a group may lead to significant discriminatory consequences with cascading negative well-being outcomes). Directing available cognitive resources to these judgments may thus reflect a costly error management strategy among marginalized group members, whereas for dominant group members, errors are likely to be less costly and cognitive resources more available. It is thus possible that the outcomes of the application stage of people perception processes may be similar among dominant and marginalized group members, but the underlying mechanisms deserve further attention.

People perception has emerged relatively recently as a new frontier in social vision research (see Alt & Phillips, 2021). We believe that this emerging area has particular implications for marginalized group members, and call for researchers in this area to adopt paradigms which center marginalized perceivers. Given discrimination can and does occur at multiple levels, including intraindividual (i.e., between individuals), interpersonal (e.g., organizations and communities), and institutional levels (e.g., legal systems; see Matsick et al., 2021) we believe that beyond the contributions of person perception research to understanding the intraindividual dimension of discrimination, people perception research has the potential to develop successful interventions to target the interpersonal level. Further understanding, and developing interventions to intervene upon, prejudice at this level will contribute to more positive outcomes for marginalized group members, particularly if their perspectives are centered in these developments. Centering marginalized perspectives also has the power to elucidate novel realms of inquiry under the social vision umbrella, such as the perception of *social objects*.

#### Perception of social objects

Stimuli which are nonsocial in nature (i.e., not people, faces, or bodies) can convey significant social meaning to people with marginalized identities, but may not communicate social information to people with dominant identities. Such stimuli, sometimes called “ambient cues” (Cheryan et al., 2009) have often been examined through the lens of social identity threat; beyond representation of diversity as discussed prior, objects such as posters, bathroom signs, and even chairs can signal identity threat or safety in a given space (Chaney & Sanchez, 2018; Cheryan et al., 2009; Oswald et al., 2022 see Kruk & Matsick, 2021 for review). For example, nonstereotypical posters depicting nature scenes increase women’s interest in computer science (a stereotypically male domain) relative to stereotypically male (Star Trek) posters (Cheryan et al., 2009), and large chairs without arms communicate belonging to fat people while small chairs provoke identity threat (Oswald et al., 2022).

We consider these objects to be *social objects* because they take on a distinct social meaning for marginalized group members by conveying threat or safety/belonging on the basis of their social identity. We also consider broader environments and spaces (e.g., architecture and rooms) as social objects; as Xiao and colleagues (2016) review, a number of features of environments, including space and distance, are shaped by top-down influences including those rooted in identity. That perception of objects is shaped by the social context surrounding the object, as well as characteristics of the perceiver, is not a novel concept (e.g., Biederman et al., 1982; Boyce & Pollatsek, 1992). However, this concept has not been fully integrated with social identity perspectives, which highlight the social nature of objects and the messages they convey to perceivers. We propose an integration of these disparate perspectives has the potential to illuminate a new frontier for social vision work, bring new methodological approaches to bear on the social identity tradition, and provide a theoretical backdrop for developing conceptual models of social identity contributions to object perception.

Cross-cultural work again highlights the ability of one’s identity to shape what we see when viewing objects or environments. Just as cultural differences have been found for the processing of faces, and crowds of faces, Westerners have been found to be relatively more biased toward focal information and Easterners toward global information when viewing objects (e.g., Masuda & Nisbett, 2001) and scenes (e.g., Masuda & Nisbett, 2006). Work in this domain reveals that East Asian participants pay more attention to contextual cues in the environment than do Western participants. This bias in visual attention also influences cultural differences in the spaces that we build around ourselves. In one study, visual scenes were selected from Japan and the United States (e.g., schools, hotels), and it was found that the scenes from Japan were more complex with greater contextual features than U.S. scenes (Miyamoto et al., 2006). In a follow-up study, they found that exposure to these scenes likewise influenced Japanese and U.S. participants’ attunements to focal versus holistic information, suggesting that we create visual environments to reside within that both reflect our cultural identity and prime distinctive patterns of visual perception.

The social signals objects and environments send may also be perceived differently depending on the identities of perceivers and the social location of these identities. For example, historic antebellum buildings are perceived more negatively by Black people than by White people, and Black people report feeling less welcome than their White counterparts in these buildings (Driskell & Trawalter, 2021). This may be because Antebellum architecture serves as a signal of racism and slavery—and thus threat—for Black, but not White people (Driskell & Trawalter). Additional research has demonstrated that, among autistic people, relative to neurotypical people, hominess in a space—that is, how much a space feels personal—is associated with approach decisions, suggesting that hominess serves as safety cue for this group, perhaps due to a preference for familiarity among autistic people (Vartanian et al., 2021).

Considering the environment, and features thereof, as social objects requires centering the perspectives of marginalized group members, as these objects do not hold the same identity-relevant meanings for dominant group members; in fact, due to their lack of vigilance to such cues, dominant group members may not even notice them (see Kruk & Matsick, 2021). Marginalized group members are attentive to these cues because of their social location, which renders them vigilant to identity threats and, correspondingly, cues to safety (Meyer, 2003; see also Kruk & Matsick, 2021). The affordances of a space or object are thus linked to the perceiver of that space or object, as well as the perceiver’s motivations (e.g., to seek safety; Meagher & Marsh, 2017). Who is allowed to realize the affordances of different objects and places is determined by social identities, and systems of oppression may reinforce limitations on marginalized individuals’ ability to realize certain affordances, particularly when objects or places are oriented to serve dominant groups (see Meagher, 2020).

Research on what we refer to as *social objects* has yet to adopt a vision science approach. However, we argue this marriage could be particularly fruitful for advancing the science of social vision. Specifically, understanding how marginalized perceivers direct visual attentional resources to, and mentally represent, visual cues to social identity threat (i.e., threat and safety cues), and how these processes differ or are similar to those among dominant group members, could help to elucidate the potential disproportionate cognitive burden of visual attention to social objects on marginalized group members, consequences of this attentional orienting, and how these processes differ or are similar to those for traditional social stimuli. For example, based on previous theorizing regarding differential vigilance among dominant and marginalized group members (Kruk & Matsick, 2021), we theorize that marginalized group members would be more likely to direct visual attention to threat (and safety) cues than would dominant group members, and that this would reduce marginalized group members’ capacity to attend to other tasks or stimuli in that context. In essence, we propose a novel visual pathway for the noted attentional effects of exposure to threat cues (e.g., Meyer, 2003). Social vision methodologies such as eye-tracking could help to elucidate this pathway and increase the range of stimuli considered by the field of social vision. These innovations can develop only by orienting toward marginalized perspectives.

## Theoretical Implications of Centering Marginalized Perspectives in Social Vision

Prioritizing marginalized perspectives in people perception research has the potential to make significant contributions to theory. In particular, this approach may assist in elucidating boundary conditions and identity-based moderators of effects currently considered to be generalizable, and in developing new theoretical accounts which are inclusive of more diverse experiences. This is true of work in the domains of person, people, and social object perception.

For example, a recent study of ensemble coding among autistic children revealed that the pervasive deficits in emotion recognition typically associated with autism symptomatology were absent in an ensemble discrimination task; autistic children were not only able to detect ensemble emotion with the same degree of accuracy but also used similar visual processing strategies to their typically developing peers (Karaminis et al., 2017). This challenge to long-standing understandings of emotion recognition in autism hints at the potential for novel theoretical developments to emerge when marginalized perspectives are centered in work on people perception. Furthermore, work prioritizing the perspectives of marginalized group members can serve to counter certain theories. For example, Bogart and Matsumoto’s (2010) study of facial expression recognition in people with Moebius syndrome—which produces complete or nearly complete facial paralysis—indicated that people with Moebius syndrome did not differ from controls in emotion recognition accuracy, countering the notion that facial mimicry, which cannot occur in Moebius syndrome, is necessary for facial expression recognition.

These examples, while not exhaustive of relevant work, demonstrate the capacity of social vision research that prioritizes marginalized perspectives to challenge dominant theoretical assumptions and to generate new theoretical accounts of perceptual processes which are not rooted exclusively in the psychology of dominant group members. We encourage researchers to consider the theoretical value of including marginalized group members in their research. As one excellent example of this type of theoretical accounting, we point readers to Hinzman et al.’s (2022) review on cross-race effects in face memory, which delineates proposed moderators of these effects as they relate to the identities of marginalized perceivers.

## Applied Implications: Translating Social Vision to Social Action

We have theorized a number of important ways in which prioritizing and centering marginalized group perspectives would contribute to our understanding of the intersection between perceptual processes and intergroup relations, including theoretical developments toward generalizability. Beyond a more generalizable and comprehensive scientific understanding of these phenomena, we believe that the recommendations and directions outlined here have the capacity to unleash the translational potential of social vision research.

Specifically, we believe social vision research could be harnessed to improve outcomes (e.g., health disparities) among marginalized group members if it were to attend to marginalized perceivers. To date, social vision research aiming to improve health and well-being outcomes among marginalized group members has primarily focused on understanding how dominant group members’ perceptual processes underlie expressions of stereotyping and prejudice, and point to prejudice-reducing interventions targeted toward these processes (e.g., reducing prejudice toward fat people through visual adaptation to fat bodies; see Lick, 2015). As experiences of prejudice and stigmatization predict poor physical and mental health outcomes among a wide range of marginalized groups (e.g., Hatzenbuehler et al., 2013; Link & Phelan, 2006), intervening upon prejudice and stigmatization is a natural target for improving the health outcomes of marginalized groups; however, work aiming to intervene on the dominant group’s perceptions may be missing half of the equation.

Work on intergroup variability among marginalized group members demonstrates that whether and how one perceives experiences of stigmatization and prejudice is a significant, meaningful contributor to health outcomes. For example, Figueroa and Zoccola (2015) found that sexual minority individuals high in stigma consciousness reported significantly greater psychological distress and worse health outcomes, with the association between stigma consciousness and self-reported health being fully mediated by perceived discrimination. Therefore, it is not only how dominant groups perceive marginalized group members that contributes to poor health outcomes in intergroup contexts, but also how marginalized group members experience intergroup processes. Furthermore, existing social vision research fails to address prejudice that emerges outside of direct intergroup contexts (e.g., institutionalized prejudice reflected through physical barriers to accessing spaces; see Oswald et al., 2022).

Understanding how marginalized group members perceive social environments—particularly as potential sources of stigma, identity threat, or discrimination—can contribute to the development of more relevant interventions which acknowledge both intergroup heterogeneity among marginalized group members and the numerous ways in which marginalization can be experienced both within and outside of direct intergroup contact (see Matsick et al., 2021). For example, drawing upon our theorizing of social objects, we suggest that using social vision methodologies such as eye tracking to understand how marginalized group members visually process environmental cues to identity threat and safety could be a fruitful direction for future research. For example, one could examine how fat people visually navigate the threatening environment of a doctor’s office and search for cues to their belonging in that space. Visual attention to a scale, or fixations on a poster about healthy diet, might be related to experiences of social identity threat (see also Oswald et al., 2022), and the time spent looking at these objects may, for example, predict a worse interaction with the health care provider (e.g., due to compounding psychosocial effects of social identity threat such as heightened anxiety), resulting in poor health outcomes for the fat patient.

We imagine that such a line of research could assist in identifying which identity cues in the environment serve as salient attractors of visual attention, under what conditions marginalized group members orient to threat and safety cues, how long they spend looking at these cues, how looking time influences relevant outcomes such as sense of belonging, and what marginalized group members may be missing (relative to dominant group members) when their visual attention is directed to these cues. Information gleaned from such studies could be used to develop interventions aiming to improve applied outcomes such as increasing sense of belonging or reducing identity threat among marginalized group members. In the example above, such interventions might include moving scales to a non-visible area of the office and ensuring that posters on the wall highlight inclusive messaging. Such interventions, when centered on marginalized perceivers, have the potential to meaningfully improve health outcomes (see also Kruk & Matsick, 2021). Our commitment to this potential is reflected in the following recommendations for engaging marginalized groups in psychological science both within and beyond social vision research.

## Recommendations For and From Social Vision

Above, we provide recommendations directed toward researchers for engaging marginalized groups in three specific areas of social vision research: person perception, people perception, and social object perception (see Table 2). In this section, we take a wider lens, building upon our theorizing to summarize key takeaways from this work directed toward those working both within and outside of the social vision sphere. These recommendations are intended to develop the field such that it can approach the theoretical and translational impact outlined in the preceding sections.

Here, we provide specific, actionable recommendations for academics working both in the capacity of researchers and as journal editors and reviewers, and provide examples where available of admirable execution of these recommendations in social vision research. Beyond researchers, we focus on editors and reviewers given the peer-review process acts as a gatekeeper for the information entering academic knowledge, which instills editors and reviewers with significant power over the knowledge generation process. Table 3 summarizes key recommendations for these groups.

**Table 3.** Key Takeaways and Recommendations for Action: Researchers and Editors/Reviewers Within and Beyond Social Vision.

Target group	Key takeaway	Recommendations	Examples of application
Researchers	Include marginalized group members in study generation and design.	Work with individuals with marginalized identities to develop research that prioritizes their experiences and needs. Develop and disseminate research in ways accessible to broader communities. Train and retain marginalized psychologists.	MacLennan et al. (2022) have an autistic author on the project team and sought feedback from members of the autism community in their study of sensory experiences
	Consider alternative methodologies.	Consider alternative approaches to quantitative/experimental paradigms, especially qualitative, critical, and participatory action research. Read widely on these methodologies and consider collaborating with experienced colleagues and members of marginalized groups to execute these frameworks effectively.	Friedman (2012, 2016) effectively uses qualitative methods to understand blind people’s perception of race and gender
	Generalize from, not just to, marginalized groups.	Conduct work that generates knowledge based on marginalized group members’ experiences. Take seriously marginalized group members’ accounts of their experiences and consider how these could contribute to a more robust psychological science.	Adetula et al. (2022) theorize how African people’s psychological experiences may generalize to cultures outside of Africa, and how incorporating African perspectives can generate a more inclusive psychology
Editors/reviewers	Recognize the generative capacity of work focused on marginalized groups.	Encourage special issues focused on marginalized groups and their experiences. Ensure that journal submission guidelines reflect the value placed on this kind of work.	*Sociological Spectrum* ran a special issue dedicated to “intersectional experiences and marginalized voices” (see Donley & Johnson, 2021)
	Question the assumption that majority group members must be included to produce generalizable knowledge.	Institute overall journal policies regarding addressing generalizability and ensure that these requirements are standardized regardless of the nature of a specific sample. Provide theoretical arguments for including majority controls where appropriate.	*Psychological Science* requires explicit discussion of generalizability and sample justification (see Association for Psychological Science, 2022)
	Include marginalized group members as members of the editorial board and as reviewers.	Encourage diversity of individuals involved in the editorial and review process. Make data on this facet of diversity available. Consider who is reviewing work on marginalized group members and what perspectives they bring to the work.	*The Clinical Neuropsychologist* successfully enhanced editorial diversity through education, implementation, and accountability efforts (see Taylor & Francis, n.d.)

### Recommendations for Researchers

#### Include marginalized group members in study generation and design

Perhaps the most critical aspect of developing a psychological science more attuned to marginalized groups is their own involvement in the development of that science. Marginalized group members bring to the table incredibly valuable experiential knowledge which is inaccessible to dominant group members (Haraway, 1988; Harding, 2004). For example, heterosexual people cannot know what it is like to live every day as a person with a sexual minority identity, what it is like to face the repeated task of “coming out” every time a new audience is encountered, or what it is like to face microaggressions and explicit prejudice when out with a same-sex partner. People with marginalized identities have unique perspectives on how these intergroup relations occur (see Matsick et al., 2022; Shelton, 2000).

To bring these perspectives into the canon of psychological science, these perspectives must be valued and engaged with in study generation and design. Research questions should be developed by, or in tandem with members of relevant marginalized groups. For example, when seeking to study how autistic people perceive their social environments, people with autism should be included in the process of conceptualizing and designing the study. Autistic people (and people with other marginalized identities) may be able to identify areas of their lives in which intervention would be helpful, and point to specific issues which are salient in their lives and could benefit from scientific exploration. In some cases, marginalized group members can provide their own perspective on what aspects of the environment are salient, what social information attracts their attention, and how this influences their socioemotional experiences (see MacLennan et al., 2022).

Involving marginalized group members in these decision-making processes will lead to a psychological science with greater impact and relevance for these individuals, and therefore better achieve the theoretical and translational goals of psychological science. Efforts in this direction could include strategies such as increasing community involvement in the research process through increased interaction and community-based dissemination of information (e.g., holding open, accessible research panels to receive feedback from members of the community; disseminating research findings in accessible ways such as through email or brochures, or through participation in podcasts or other media dissemination formats; see also Buchanan et al., 2021; Matsick et al., 2021), through the use of alternative methodologies which emphasize participatory action (see recommendation 2), and through efforts to train and retain marginalized group members as researchers (see also Adetula et al., 2022; American Psychological Association 2021).

#### Consider alternative methodologies

Social vision research, like most psychological research, traditionally relies on quantitative paradigms. These paradigms can be useful for answering certain questions, but can also hinder theoretical development and contribute to the ongoing resistance to intersectionality in psychology (see Matsick et al., 2021; Settles et al., 2021; Shields, 2008). Innovative uses of qualitative approaches, particularly those in the vein of participatory action research, could be especially useful in prioritizing marginalized perspectives to improve the translational impact of social vision research. These methodological frameworks seek to redress power inequities in the research process by involving research “participants,” often marginalized group members, as co-researchers, who participate in the development of projects and direct these projects toward relevant research questions, methodologies, and action outcomes (see Dupont, 2008; Lykes & Crosby, 2014; Ponic et al., 2010). Such approaches are compatible with intersectional theorizing, and even necessitate attention to intersecting relations of power as they shape the research process and the lived experiences of the co-researchers (see Lykes & Crosby, 2014). The rich history of feminist psychological work provides excellent guidance particularly for conducting qualitative and intersectional psychological research (e.g., Buchanan & Wiklund, 2021; Cole, 2009; Else-Quest & Hyde, 2016a, 2016b; Grzanka et al., 2017; McCormick-Huhn et al., 2019).

Some researchers—many who may not be considered canonically social vision researchers—demonstrate the capacity of qualitative methods to prioritize marginalized perspectives in social vision. For example, sociologist Asia Friedman uses qualitative interviews to examine blind people’s perceptions of race (Friedman, 2016) and gender (Friedman, 2012), fruitfully delineating how blind people—including those blind from birth—rely on visual understandings of race and gender, similarly to their sighted counterparts. This work exemplifies a number of the recommendations we endorse in the current paper, including forwarding marginalized voices, allowing marginalized people to dictate the aspects of the research question which were salient to them, and highlighting similarities, and not just differences, between blind and sighted people. Similarly, recent research on the sensory experiences of adults with autism used a participatory action framework to engage these adults in co-developing the research design, allowing adults with autism to explore and understand the aspects of their sensory experiences—including visual experiences—which are most salient to them (see MacLennan et al., 2022). In doing so, this work allows marginalized participants/co-researchers to focus on issues relevant to them, and gains greater insight into autistic adults’ visual experiences unconstrained by neurotypical ideas about these experiences. We recommend that researchers consider adopting frameworks which allow marginalized group members the agency to focus on issues relevant to their experiences. To do so, researchers unfamiliar with these methods may consider reading widely (within and beyond psychology) on these methodological frameworks (see also Buchanan & Wiklund, 2021) and collaborating with colleagues who do have experience in these methodologies, likely those from other areas of psychology (e.g., community psychology) or other disciplines which more frequently utilize these methodologies (e.g., sociology). In addition, researchers should take seriously the notion of collaboration with members of the marginalized group they intend to study.

Furthermore, though we focus primarily on the visual system as a conduit for social information, it is important to underscore here that social factors are expected to impact perception more broadly, and our recommendations extend to this broader sense of perception. Research in the domain of social vision has revealed that humans are not simple click-and-whir perceptual machines; perception is constructed, and thus all social sensory inputs (i.e., vision, audition, touch etc.) are likely shaped through the lens of the perceiver. The same social factors detailed in this paper are expected to shape other perceptual experiences, including multisensory perception, and alternative methodologies for examining the effects we describe here (e.g., social identity threat effects) may adopt multisensory approaches or non-visual approaches. This might include, for example, understanding fat people’s experiences of identity threat when they walk on a squeaky floor, or understanding how blind people perceive race through auditory cues. This broader conception of social vision and of perceiver effects on perception can widen the lens of social vision research and contribute to a more inclusive understanding of human psychology.

#### Generalize from, not just to, marginalized groups

Psychological research on marginalized group members often examines how they deviate from dominant group members, or whether theories developed with dominant groups generalize to marginalized groups (e.g., testing whether Western psychological theories generalize to African people, who are marginalized in psychological research; see Adetula et al., 2022). As Adetula et al. argue, these attempts to understand non-WEIRD groups by *generalizing* dominant psychological theories—which are based on ideas and data from dominant group members—rather than *generating* theories and knowledge from marginalized perspectives, creates significant gaps in our understanding. Furthermore, this approach of testing dominant theories on underrepresented groups tends to generate knowledge which primarily benefits the dominant group (Adetula et al.). We extend this argument to apply not only to groups marginalized in the psychological literature (e.g., African people) but to marginalized groups more broadly.

Psychological research suffers from the pervasive sentiment that research centered on marginalized groups is not generalizable and therefore cannot make a significant contribution to theory (Avery et al., 2022). However, it is possible and likely that these groups experience psychological processes that could provide insight into the complexity of the human experience, regardless of whether these processes are similar to or different from those of the dominant groups who comprise the bedrock of dominant psychological theorizing (see also Adetula et al., 2022). For example, understanding how colorblind people visually experience the world led to developments in scientific understandings of color perception more broadly; the discovery of the wavelengths of light which differentiate colors, in tandem with the knowledge that some people perceive color differently (or lack the perception of some colors altogether) prompted the scientific discovery of the retina’s color receptors (Rogers, 2021), which have important implications for social vision. For example, color vision is linked to the perception of emotion expression and to human skin tone, playing an important role in interpersonal perception (Changuzi & Shimojo, 2010).

Understanding how visual and other psychological processes occur among marginalized groups can contribute to the development of generalizable theories, in addition to highlighting boundary conditions in existing theoretical accounts. Furthermore, understanding psychological processes among marginalized group members can contribute to the development of a psychological science that holds relevance to everyone (Adetula et al., 2022). We thus recommend that researchers should take seriously marginalized group members’ accounts of their experiences and consider how these accounts could be generalized to other groups, including dominant groups. When developing theoretical accounts or testing the generalizability of theories, researchers should also question who the work benefits—does the knowledge that is generated serve the relevant marginalized community? Researchers should consider consulting with members of the relevant marginalized group to answer this question, as those with experiential knowledge of marginalization will best be able to indicate whether certain knowledge is relevant to their experience.

### Recommendations for Editors and Reviewers

#### Recognize and value the generative capacity of work focused on marginalized groups

Research on marginalized group members is often sequestered to “specialty journals” (Buchanan et al., 2021), in part due to a pervasive notion noted above that research centered on marginalized groups is not generalizable and therefore cannot make a significant contribution to theory (Avery et al., 2022). However, as noted above, these sentiments are misguided and ignore the generative capacity of this work, both in terms of developing new psychological theories and for testing the boundary conditions of WEIRD psychological theories. Those with the power to do so should, particularly those with editorial power, should encourage submission of research centered on marginalized group members to larger, more mainstream, and general journals, including through explicit statements in journal aims and scope which reflect the value of this work and through the provision of special issues on relevant topics (see also Buchanan et al., 2021). Editors may also consider opening their journals to a broader range of submissions to introduce greater diversity and potential for representation of marginalized perspectives, for example, by considering both qualitative and quantitative research.

#### Question the assumption that majority group members must be included to produce generalizable knowledge

The failure of psychological science to generalize *from* marginalized groups is often enforced through the peer-review process. Reviewers and editors often request for research focusing on marginalized groups to include a dominant group comparison to be considered “generalizable” and therefore publishable, while work on dominant groups is not held to this same standard (i.e., requiring a marginalized comparison group; Avery et al., 2022; Buchanan et al., 2021). Comparison to a dominant group should be pursued only when theoretically necessary, and the burden of proof for this relevance should fall to reviewers and editors rather than authors (see also Buchanan et al., 2021; Matsick et al., 2021). Requests for between-group analyses (i.e., tests of group differences) within a sample should be held to these same standards (see also Buchanan & Wiklund, 2021). Editors/journals should have policies for addressing these issues with reviewers directly (e.g., reviewer “training” modules which instruct reviewers when it is appropriate to request dominant group controls). Furthermore, journals should outline specific policies regarding how authors should address issues of generalizability within their manuscript and manuscript title (e.g., requiring constraints on generality statements; see Simons et al., 2017), and these policies should be applied uniformly regardless of a given paper’s subject matter or sample (see also Avery et al., 2022; Buchanan et al., 2021). Such policies would reduce biases embedded in the peer-review process which require disproportionate justification of marginalized samples.

#### Include marginalized group members as members of the editorial board and as reviewers

Scholars with marginalized identities are less frequently involved in the review process, particularly with mainstream outlets; work in those outlets thus continues to be evaluated predominantly by majority group members (Avery et al., 2022; Buchanan et al., 2021). Editors should ensure that their editorial boards and ideally their reviewers reflect the diversity of work published in the journal. Journals should also make accessible data on the diversity of their editorial boards and reviewers. As Avery et al. suggest, journal editors could also consider “matching” submitted manuscripts on marginalized groups to reviewers with relevant expertise—especially experiential expertise—on those topics, either manually or with the use of algorithms, and can also encourage researchers to submit recommended reviewers and select editors with relevant expertise. It is important to note that women, racial minorities, and those with additional marginalized identities already bear a disproportionate burden of service work in academia (e.g., Malisch et al., 2020; Miller & Roksa, 2020); editors should therefore consider allocating compensated roles or honorariums to marginalized scholars for their contributions—thereby balancing acknowledgment of these disproportionate burdens with the need for marginalized perspectives. Buchanan et al. (2021) provide additional, excellent suggestions for reducing racism in the conduct and dissemination of psychological science, many of which would benefit a variety of marginalized groups if implemented.

## Constraints on Generality

Our theorizing draws upon literature from a range of disciplines, geographical areas, populations, cultures, and perspectives. To the extent possible, we have sought to represent a variety of perspectives in the current work; however, we are limited by the existing literature and its predominant focus on dominant group perceivers. Many of the empirical works we cite do not provide comprehensive demographic information, and focus only on singular identities of interest. Furthermore, by focusing primarily on literal vision throughout the manuscript, we tend to cite work focused on this understanding of vision and therefore our theorizing is constrained to those with typical visual processing capacities. The theoretical framework used here to conceptualize social vision, however, can apply to other forms of social sensory perception (e.g., audition) and multisensory perception more broadly, which were beyond the scope of this review. Finally, we believe that the field of social vision is constrained in its generality by its predominant focus on dominant group perceivers, and hope that the suggestions we provide in the current work can contribute to the development of an unconstrained social vision with a greater capacity for generality.

## Conclusion

Although existing social vision research provides a wealth of knowledge regarding how marginalized group members are perceived, much less is known about how marginalized group members perceive their worlds. Taking a feminist approach of empowering marginalized groups as active members of the research process and as informants of (necessarily bidirectional) intergroup relations (see also Matsick et al., 2022; Shelton, 2000) will reveal how perceptual processes shape intergroup relations from both sides, and how marginalized group members may differentially experience their perceptual worlds. These understandings can be harnessed to develop evidence-based interventions to improve well-being among marginalized group members that work alongside traditional interventions aimed at understanding and changing dominant group behavior. We believe in the capacity of this approach to generalize to members of diverse marginalized groups and to extend to those with multiply marginalized identities. An approach to social vision which prioritizes marginalized group perspectives will lead to translational developments which prioritize these perspectives and are thus better able to serve the needs of marginalized group members.
